# The production of isoprene from cellulose using recombinant *Clostridium cellulolyticum* strains expressing isoprene synthase

**DOI:** 10.1002/mbo3.1008

**Published:** 2020-02-28

**Authors:** Christian Janke, Stefan Gaida, Stefan Jennewein

**Affiliations:** ^1^ Fraunhofer-Institut für Molekularbiologie und Angewandte Ökologie Aachen Germany

**Keywords:** green chemicals, metabolic engineering, synthetic rubber, terpenoids

## Abstract

Isoprene is an important bulk chemical which is mostly derived from fossil fuels. It is used primarily for the production of synthetic rubber. Sustainable, biotechnology‐based alternatives for the production of isoprene rely on the fermentation of sugars from food and feed crops, creating an ethical dilemma due to the competition for agricultural land. This issue could be addressed by developing new approaches based on the production of isoprene from abundant renewable waste streams. Here, we describe a proof‐of‐principle approach for the production of isoprene from cellulosic biomass, the most abundant polymer on earth. We engineered the mesophilic prokaryote *Clostridium cellulolyticum*, which can degrade cellulosic biomass, to utilize the resulting glucose monomers as a feedstock for the production of isoprene. This was achieved by integrating the poplar gene encoding isoprene synthase. The presence of the enzyme was confirmed by targeted proteomics, and the accumulation of isoprene was confirmed by GC‐MS/MS. We have shown for the first time that engineered *C. cellulolyticum* can be used as a metabolic chassis for the sustainable production of isoprene.

## Graphical abstract

We engineered the mesophilic prokaryote *Clostridium cellulolyticum*, which can degrade cellulosic biomass, to utilize the resulting glucose monomers as a feedstock for the production of isoprene. We have shown for the first time that engineered *C. cellulolyticum* can be used as a metabolic chassis for the sustainable production of isoprene.

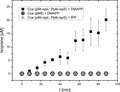

## 1 INTRODUCTION

Isoprene or 2‐methyl‐1,3‐butadiene (C_5_H_8_) is a volatile chemical used mainly for the production of synthetic rubber. There is a strong industrial demand for isoprene, with an annual production volume of up to one million tonnes (McCoy, 2008). Most isoprene is still isolated as a by‐product during the thermal cracking of oil or naphtha and the changeover to sustainable production from renewable sources has yet to be achieved. However, many plant species express the enzyme isoprene synthase (IspS) and up to 500 million tonnes of isoprene is released naturally by plants every year (Guenther et al., 2006). IspS converts dimethylallyl diphosphate (DMAPP), which is synthesized together with its isomer isopentenyl diphosphate (IPP) via either the mevalonate (MVA) or 1‐deoxy‐D‐xylulose 5‐phosphate (MEP) pathways in all living organisms, into isoprene via an elimination reaction (Koksal, Zimmer, Schnitzler, & Christianson, 2010)**.**

The industrial production of volatile chemicals such as isoprene is difficult to achieve in plants, but the corresponding enzymes have been transferred to the bacterium *Escherichia coli* and yeast. *Escherichia coli* cells expressing a heterologous IspS, isopentenyl diphosphate isomerase (Idi), and the MVA pathway produce up to 24 g of isoprene per liter of culture medium with glucose as the carbon source (Yang et al., 2016). However, glucose is derived from food and feed crops, which means there is increasing competition between the food/feed and chemical industries for agricultural land and resources. Ideally, isoprene should be produced from a renewable waste stream such as cellulose, which is the most abundant polymer on earth.

To unlock the potential of this feedstock, we selected the mesophilic and strictly anaerobic bacterium *Clostridium cellulolyticum* (Petitdemange, Caillet, Giallo, & Gaudin, 1984), which can break down cellulosic biomass to provide carbon and energy. We used this model microbe as a metabolic chassis for the expression of the poplar gene encoding IspS as an initial and crucial step toward isoprene production at industrial scale. The substrate DMAPP is supplied by the endogenous MEP pathway in *C. cellulolyticum*. A combination of targeted proteomics and GC‐MS/MS was used to confirm the expression of active IspS and the accumulation of isoprene. This is the first demonstration of isoprene production by recombinant cellulolytic bacteria and lays the foundation for a sustainable isoprene production process based on lignocellulosic biomass.

## 2 MATERIALS AND METHODS

### 2.1 Cloning

For all experiments, we used the previously described shuttle plasmids pIM and pM9 (Gaida, Liedtke, Jentges, Engels, & Jennewein, 2016). The *Populus alba ispS* gene (GenBank accession number: Q50L36) was modified to delete the first 51 codons and to introduce the mutation P494L. The sequence was then codon‐optimized by ATG:biosynthetics GmbH (Germany) and cloned downstream of the *C. acetobutylicum* phosphotransbutyrylase (*ptb*) promoter (Tummala, Welker, & Papoutsakis, 1999) using the Gibson Assembly process (Gibson et al., 2009) with the following overlaps: forward 5′‐GTACCGGTGGTGGCTCCGGTGATGACGACGACAAG‐3′ and reverse 5′‐GGCTTTGTTTAGCAGCCTAGGTATTAATCAATTAG‐3′. The *E. coli* BL21 (DE3) *idi* gene was cloned downstream of the thiolase promotor in the modified vector pSOS95 (GenBank accession number: AY18768) by restriction digest and ligation. The constructs were named pIM‐repL::P_ptb_‐ispS and pSOS95::P_thl_‐Idi_Ec, respectively (Table 1). We used *E. coli* NEB10B cells (New England Biolabs GmbH, Germany) for cloning.

**Table 1:** Listing of plasmids and strains

Plasmid and strains	Description	Reference
pIM‐repL::Pptb‐ispS	The *ispS* gene was cloned downstream of *C. acetobutylicum* phosphotransbutyrylase (ptb) promoter. Plasmid backbone (pIM) has recently been published (Philipps et al., 2019)	This study
pM9	Control plasmid	Gaida et al. (2016)
pSOS95::P_thl_‐idi_Ec	The gene for the isopentenyl diphosphate isomerase is under control of thiolase promoter from *C. acetobutylicum*	This study
Cce	*C. cellulolyticum* strain H10 (DSM 5812; DSMZ, Braunschweig, Germany)	Petitdemange et al. (1984)
Cce (pIM‐repL::Pptb‐ispS)	Cce strain for episomal expression of the *ispS* gene	This study
Cce (P_ptb_‐ispS)	Cce strain for expression of the genomically integrated *ispS* gene	This study
Cce (pM9)	Cce strain with control plasmid	This study
Cce (P_ptb_‐ispS, pSOS95::P_thl_‐idi_Ec)	Cce strain for the expression of the genomically integrated *ispS* gene and of the plasmid encoded *idi* gene	This study

### 2.2 Cultivation and electroporation of *Clostridium cellulolyticum* and genomic integration

The *C. cellulolyticum* strain H10 (DSM 5812; DSMZ, Braunschweig, Germany) was cultivated essentially as previously described (Gaida et al., 2016), but only 6 g/L of crystalline cellulose was provided as a carbon source. Following electroporation (Gaida et al., 2016), genomic integration of the P_ptb_‐ispS expression cassette was achieved by adding 0.5% xylose to induce the expression of Himar1 transposase under the control of a xylose‐dependent promoter as previously described (Philipps, Vries, & Jennewein, 2019). The resulting strain was named Cce(P_ptb_‐ispS) (Table 1).

### 2.3 GC‐MS/MS and IspS activity assay

GC‐MS/MS experiments were carried out using the TQ8030 system (Shimadzu) fitted with a headspace syringe (Gerstel) and ZB624 column (Phenomenex). We used toluene (0.001%, dissolved in acetone) as an internal standard, and isoprene concentrations were determined against external standards. The 68 > 67 Da transition was used for quantification, assuming that 100% of the isoprene was found in the gas phase. Multiple reaction monitoring (MRM) was used to quantify substances with transitions of 68 > 67 and 67 > 41 Da for isoprene and a retention time of 2.2 min. The GC‐MS/MS system was held at 40°C for 1 min, followed by a gradient of 40°C/min to reach a plateau at 200°C for 8 min. For the activity assay, cells were harvested after cultivation for 72 hr (early stationary phase) and were disrupted at 4°C with a sonicator (Hielscher). The quantity of total soluble protein was determined using a Bradford assay (Bio‐Rad). Enzyme activity was determined by mixing 50 µg of total protein and 5 µg DMAPP with sufficient buffer (50 mM Tris‐HCl, 10 mM MgCl_2_, pH 8) to reach a total volume of 500 µl in 10‐ml headspace tubes. The headspace was sampled at 8 min intervals following the addition of DMAPP and 12 samples were taken in total, each in triplicate.

### 2.4 Targeted proteomics

Targeted proteomics was carried out as previously described (Stergachis, MacLean, Lee, Stamatoyannopoulos, & MacCoss, 2011) using either a 1200 Series LC (Agilent Technologies, USA) combined with a QTRAP 3200 MS/MS system (Sciex, USA), or a Shimadzu HPLC combined with a QTRAP 6500 MS/MS system (Sciex). Bovine serum albumin (BSA) was used as an internal control (10 ng/µl; target peptide: YLYEIAR). This was added to the crude extract before 200 µg of total protein was digested per sample. The targeted proteomics method was developed with Skyline (MacLean et al., 2010), and the peptide VDITQIK was used to detect chloramphenicol acetyltransferase (CatP, backbone marker), whereby the peptide VLSVITEPILPFER was used to detect IspS. The peptides were separated in a linear gradient by changing the ratio between buffer A (2% acetonitrile, 98% water, 0.1% formic acid) and buffer B (98% acetonitrile, 2% water, and 0.1% formic acid) from 95:5 to 5:95 with a flow rate of 0.4 ml/min.

## 3 RESULTS AND DISCUSSION

### 3.1 Plasmid cloning, transformation, and genomic integration of Isoprene synthase

The *ispS* gene was inserted downstream of the *ptb* promoter using a Gibson Assembly process. The promoter is part of a plasmid segment flanked by internal recognition sites for the Himar transposase. The plasmid DNA was transferred by electroporation into competent *C. cellulolyticum* cells and stabilized by adding thiamphenicol and clarithromycin. This episomal strain was named Cce(pIM‐repL::P_ptb_‐ispS). The plasmid DNA was reisolated and sequenced, confirming that the *ispS* gene was intact in all clones. Genomic integration was then induced by adding xylose (0.5%) to activate the transposase gene. This step eliminates the thiamphenicol resistance gene, and we confirmed that the clones were no longer able to grow in the presence of this antibiotic. This strain was named Cce(P_ptb_‐ispS), and the integrity of one clone was confirmed by sequencing.

### 3.2 Protein expression, isoprene synthesis, and in vitro IspS activity

**Figure 1:**
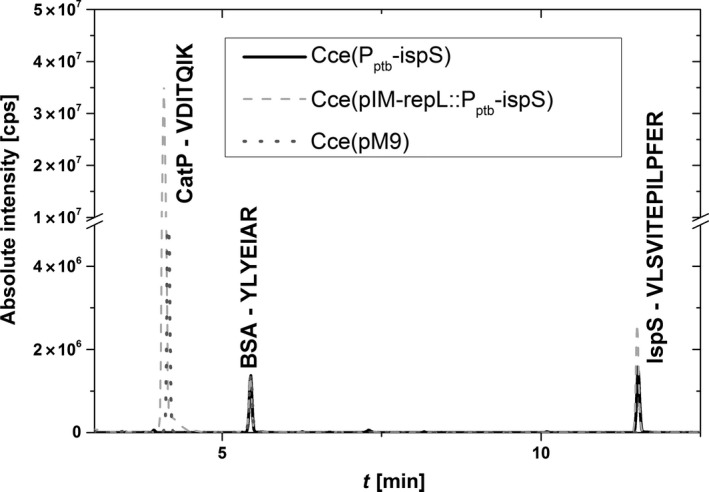
Targeted proteomics data for three recombinant *Clostridium cellulolyticum* (Cce) strains. Two multiple reaction monitoring (MRM) transitions were summed for each peptide. The detection of both transitions and the correct retention time was the minimum evidence accepted for the presence of IspS. The chromatogram of the genomic integration strain Cce(P_ptb_‐ispS) is shown in black, and is overlaid with the chromatograms of the episomal strains Cce(pIM‐repL::P_ptb_‐ispS) and Cce(pM9), which are shown with light gray dashes and gray dots, respectively. BSA was used as internal standard

The quantity of IspS protein was determined by targeted proteomics, with two MRM transitions and the correct retention time for each unique peptide taken as the minimum evidence for the presence of the target. The IspS peptide transitions were not detected in our control strain Cce(pM9) (Figure 1). However, the transitions were observed in strains Cce(P_ptb_‐ispS) and Cce(pIM‐repL::P_ptb_‐ispS), confirming that IspS was present in both (Figure 1). The genetic difference between these strains was confirmed by testing for the plasmid backbone marker CatP, which was detected only in strain Cce(pIM‐repL::P_ptb_‐ispS). BSA was added to all crude extracts as an internal standard, and there were no significant variations between different strains.

**Figure 2:**
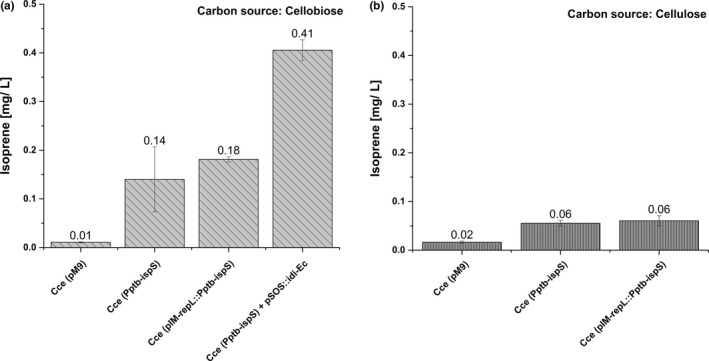
Isoprene emission by recombinant *Clostridium cellulolyticum* (Cce) cultures growing on either cellobiose (a) or crystalline cellulose (b) as a carbon source (*n* = 3). The vials were sampled using a headspace syringe before GC‐MS/MS analysis

To verify that IspS was expressed in its active form, we measured isoprene emissions after incubating batch cultures grown in headspace vials for 7 days on cellobiose (Figure 2a) or 14 days on crystalline cellulose due to the slower growth (Figure 2b). We detected higher isoprene levels for all recombinant strains compared to controls, and the cells growing on cellobiose produced up to 10‐fold more isoprene than cells growing on crystalline cellulose. Furthermore, strain Cce(pIM‐repL::P_ptb_‐ispS) produced twice as much isoprene as the genomic integrant strain Cce(P_ptb_‐ispS), probably reflecting the higher gene dosage of the episomal plasmid compared with the single‐copy integrated construct. Even so, this is a relatively small difference, probably due to a low plasmid copy number or simply the consequence of a substrate limitation. The latter assumption was supported by the results of introducing the *E. coli* gene encoding Idi, which catalyses the isomerization of IPP to DMAPP. The transformation of strain Cce(P_ptb_‐ispS) with pSOS95::P_thl_‐idi_Ec resulted in an eightfold increase in the quantity of isoprene, probably reflecting the much faster regeneration of the DMAPP pool (Lv, Xu, & Yu, 2013).

**Figure 3:**
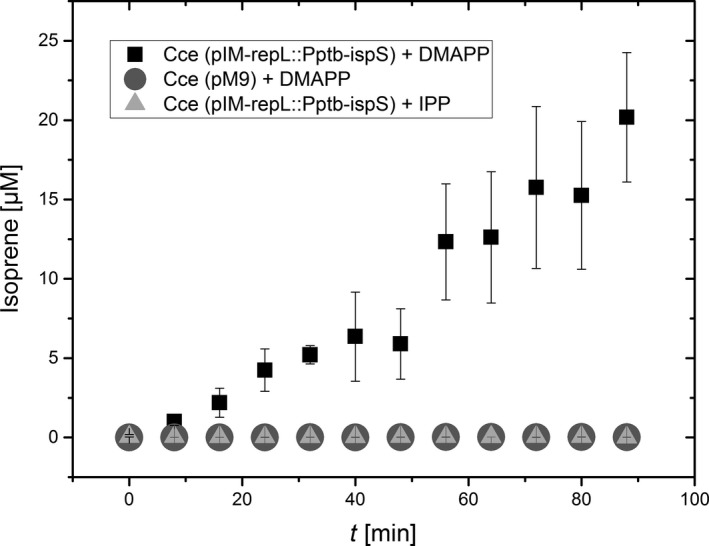
Activity assays with crude extracts and DMAPP as substrate from recombinant isoprene‐producing strain pIM‐repL::Pptb‐ispS (shown with black squares) compared with the control strain pM9 (shown with gray circles). Error bars indicate the standard deviation of three independent experiments (*n* = 3). In contrast to DMAPP, the addition of IPP to the crude extract of strain Cce(pIM‐repL::Pptb‐ispS) did not result in any isoprene formation (shown with light gray triangles)

The combined proteomics, enzyme activity, and GC‐MS/MS data provided strong evidence for the expression of an active IspS protein leading to the accumulation of isoprene, but the background levels of isoprene (≤10 µg/L) made it impossible to exclude the autocatalytic formation of isoprene from DMAPP in larger amounts in our recombinant strains. We therefore carried out in vitro enzyme tests to prove that IspS was responsible for the conversion of DMAPP to isoprene. We confirmed the DMAPP‐dependent emission of isoprene from crude extracts of the recombinant strains expressing IspS, with the extract of strain Cce(pIM‐repL::P_ptb_‐ispS) revealing activity of 0.23 µmol/min. In contrast, the recombinant control strain carrying vector pM9 (Gaida et al., 2016) revealed no background activity within the chosen time frame (Figure 3). Moreover, the addition of 5 µg IPP, which is an isomer of DMAPP but not a substrate for IspS, did not lead to any detectable activity during the experiment (Figure 3).

Overall, our combined results therefore provide strong experimental evidence for the stable expression of an active IspS in *C. cellulolyticum*. The plant enzyme is the central biological catalyst for isoprene production. Hence, its introduction into *C. cellulolyticum* is the initial and crucial step for the production of isoprene in industrial quantities. However, further steps will be necessary. Usually, the heterologously expressed MVA pathway is used for the supply of DMAPP for isoprene production in bacteria (Yang et al., 2012). Modern approaches try to integrate both pathways, the endogenous MEP and MVA pathway, to increase productivity (Yang et al., 2016). Next to well‐designed genetic constructs and proteomics, the analysis of pools of pathway intermediates will be equally important to identify the bottlenecks of isoprene production and to further evaluate *C. cellulolyticum* as a chassis for it. Therefore, a holistic approach is required for future studies to determine the actual limits of the biological system *C. cellulolyticum* for industrial‐scale production. Clostridia are often considered to be genetically difficult to access and to manipulate. Our experiments strongly indicate the stable expression of an active IspS. Thus, *C. cellulolyticum* should be considered as a well‐suited chassis for the expression of the central enzyme, the IspS from poplar, for isoprene production.

## 4 CONCLUDING REMARKS

Our work has laid the foundation for the conversion of cellulosic biomass to isoprene using metabolically engineered strains of *C. cellulolyticum*. A major limitation that hinders engineering in this species is the lack of a convenient molecular toolbox therefore preventing the quick and successful development of robust industrial production strains. To our knowledge, the work described here is the first time that an active isoprene synthase has been expressed in cellulolytic bacteria, and we observed stable expression and activity during all of our experiments. Therefore, we strongly believe that *C. cellulolyticum* is a suitable chassis for the production of isoprene from waste streams. In contrast to other renewable sources (e.g., CO_2_, utilized by photosynthetic microorganisms) cellulosic biomass has a high energy density. Another significant advantage of *C. cellulolyticum* is that growth and isoprene production depend neither on light nor the availability of oxygen. Future work should focus on engineering the MEP and MVA pathways to increase the substrate pool for isoprene synthase, as well as optimizing the utilization of cellulosic biomass.

## CONFLICT OF INTEREST

None declared.

## AUTHOR CONTRIBUTIONS

Christian Janke: involved in conceptualization (lead); investigation (equal): methodology (equal) writing—original draft (lead); writing—review and editing (lead); Stefan Gaida: involved in conceptualization (supporting); investigation (equal): methodology (equal) writing—original draft (supporting); writing—review and editing (supporting); Stefan Jennewein: involved in funding acquisition (lead).

## ETHICS STATEMENT

None required.

## Data Availability

All data generated or analyzed during this study is included in this published article.

## References

[mbo31008-bib-0001] Gaida, S. M. , Liedtke, A. , Jentges, A. H. , Engels, B. , & Jennewein, S. (2016). Metabolic engineering of *Clostridium cellulolyticum* for the production of n‐butanol from crystalline cellulose. Microbial Cell Factories, 15, 6 10.1186/s12934-015-0406-2 26758196 PMC4711022

[mbo31008-bib-0002] Gibson, D. G. , Young, L. , Chuang, R. Y. , Venter, J. C. , Hutchison, C. A. , & Smith, H. O. (2009). Enzymatic assembly of DNA molecules up to several hundred kilobases. Nature Methods, 6, 343–345. 10.1038/nmeth.1318 19363495

[mbo31008-bib-0003] Guenther, A. , Karl, T. , Harley, P. , Wiedinmyer, C. , Palmer, P. I. , & Geron, C. (2006). Estimates of global terrestrial isoprene emissions using MEGAN (Model of Emissions of Gases and Aerosols from Nature). Atmospheric Chemistry and Physics, 6, 3181–3210. 10.5194/acp-6-3181-2006

[mbo31008-bib-0004] Koksal, M. , Zimmer, I. , Schnitzler, J. P. , & Christianson, D. W. (2010). Structure of isoprene synthase illuminates the chemical mechanism of teragram atmospheric carbon emission. Journal of Molecular Biology, 402, 363–373. 10.1016/j.jmb.2010.07.009 20624401 PMC2942996

[mbo31008-bib-0005] Lv, X. , Xu, H. , & Yu, H. (2013). Significantly enhanced production of isoprene by ordered coexpression of genes dxs, dxr, and idi in *Escherichia coli* . Applied Microbiology and Biotechnology, 97, 2357–2365. 10.1007/s00253-012-4485-2 23143466

[mbo31008-bib-0006] MacLean, B. , Tomazela, D. M. , Shulman, N. , Chambers, M. , Finney, G. L. , Frewen, B. , … MacCoss, M. J. (2010). Skyline: An open source document editor for creating and analyzing targeted proteomics experiments. Bioinformatics, 26, 966–968. 10.1093/bioinformatics/btq054 20147306 PMC2844992

[mbo31008-bib-0007] McCoy, M. (2008). INDUSTRIAL BIOTECHNOLOGY Goodyear and Genencor plan biobased isoprene. Chemical & Engineering News Archive, 86, 15 10.1021/cen-v086n038.p015a

[mbo31008-bib-0008] Petitdemange, E. , Caillet, F. , Giallo, J. , & Gaudin, C. (1984). *Clostridium cellulolyticum* sp. nov., a Cellulolytic, Mesophilic: Species from decayed grass. International Journal of Systematic and Evolutionary Microbiology, 34, 155–159. 10.1099/00207713-34-2-155

[mbo31008-bib-0009] Philipps, G. , de Vries, S. , & Jennewein, S. (2019). Development of a metabolic pathway transfer and genomic integration system for the syngas‐fermenting bacterium *Clostridium ljungdahlii* . Biotechnology for Biofuels, 12, 112 10.1186/s13068-019-1448-1 31086564 PMC6507227

[mbo31008-bib-0010] Stergachis, A. B. , MacLean, B. , Lee, K. , Stamatoyannopoulos, J. A. , & MacCoss, M. J. (2011). Rapid empirical discovery of optimal peptides for targeted proteomics. Nature Methods, 8, 1041–1043. 10.1038/nmeth.1770 22056677 PMC3227787

[mbo31008-bib-0011] Tummala, S. B. , Welker, N. E. , & Papoutsakis, E. T. (1999). Development and characterization of a gene expression reporter system for *Clostridium acetobutylicum* ATCC 824. Applied and Environmental Microbiology, 65, 3793–3799. 10.1128/AEM.65.9.3793-3799.1999 10473377 PMC99702

[mbo31008-bib-0012] Yang, C. , Gao, X. , Jiang, Y. , Sun, B. , Gao, F. , & Yang, S. (2016). Synergy between methylerythritol phosphate pathway and mevalonate pathway for isoprene production in *Escherichia coli* . Metabolic Engineering, 37, 79–91. 10.1016/j.ymben.2016.05.003 27174717

[mbo31008-bib-0013] Yang, J. , Zhao, G. , Sun, Y. , Zheng, Y. , Jiang, X. , Liu, W. & Xian, M. (2012). Bio‐isoprene production using exogenous MVA pathway and isoprene synthase in *Escherichia coli* . Bioresource Technology, 104, 642–647. 10.1016/j.biortech.2011.10.042 22133602

